# Surface Nanostructuring of Parylene-C Coatings for Blood Contacting Implants

**DOI:** 10.3390/ma11071109

**Published:** 2018-06-29

**Authors:** Luigi Brancato, Deborah Decrop, Jeroen Lammertyn, Robert Puers

**Affiliations:** 1 ESAT-MICAS, KU Leuven, Kasteelpark Arenberg 10, 3001 Heverlee, Belgium; puers@esat.kuleuven.be; 2 Department of Biosystems–MeBioS, KU Leuven, Willem de Croylaan 42, 3001 Heverlee, Belgium; deborah.decrop@kuleuven.be (D.D.); jeroen.lammertyn@kuleuven.be (J.L.)

**Keywords:** Parylene-C, protein adsorption, haemocompatibility, hydrophobicity, packaging

## Abstract

This paper investigates the effects on the blood compatibility of surface nanostructuring of Parylene-C coating. The proposed technique, based on the consecutive use of O_2_ and SF_6_ plasma, alters the surface roughness and enhances the intrinsic hydrophobicity of Parylene-C. The degree of hydrophobicity of the prepared surface can be precisely controlled by opportunely adjusting the plasma exposure times. Static contact angle measurements, performed on treated Parylene-C, showed a maximum contact angle of 158°. The nanostructured Parylene-C retained its hydrophobicity up to 45 days, when stored in a dry environment. Storing the samples in a body-mimicking solution caused the contact angle to progressively decrease. However, at the end of the measurement, the plasma treated surfaces still exhibited a higher hydrophobicity than the untreated counterparts. The proposed treatment improved the performance of the polymer as a water diffusion barrier in a body simulating environment. Modifying the nanotopography of the polymer influences the adsorption of different blood plasma proteins. The adsorption of albumin—a platelet adhesion inhibitor—and of fibrinogen—a platelet adhesion promoter—was studied by fluorescence microscopy. The adsorption capacity increased monotonically with increasing hydrophobicity for both studied proteins. The effect on albumin adsorption was considerably higher than on fibrinogen. Study of the proteins simultaneous adsorption showed that the albumin to fibrinogen adsorbed ratio increases with substrate hydrophobicity, suggesting lower thrombogenicity of the nanostructured surfaces. Animal experiments proved that the treated surfaces did not trigger any blood clot or thrombus formation when directly exposed to the arterial blood flow. The findings above, together with the exceptional mechanical and insulation properties of Parylene-C, support its use for packaging implants chronically exposed to the blood flow.

## 1. Introduction

The encapsulation of electronic systems to be implanted in the human body has to ensure a hermetic seal that prevents the ingress of moisture, potentially leading to corrosion and failure of the implant. The packaging materials must be selected to minimize the risk of an adverse reaction of the organism to the foreign object. The long-term application of biomaterials in direct contact with blood represents one of the biggest challenge to the success of an implant. When a biomaterial is exposed to the blood flow, it initiates a complex series of events that begins with the adsorption of plasma proteins and can, through the adhesion and activation of platelets, lead to the formation of blood cloths or thrombi [1,2].

Several factors influence the thrombogenicity of a biomaterial. Besides its chemical composition, physical properties such as surface roughness, topography and wettability will determine the biological response of the host organism. Modifying the surface topography of a material can affect the adsorption of different proteins from the blood flow and, as a result, encourage or inhibit platelets adhesion and activation [3].

Parylene-C is a biocompatible and biostable polymer [4,5,6]. The chemical vapor deposition (CVD) process allows it to be deposited as a conformal, pin-hole free layer on any material and in any complex geometrical shape. A very thin layer of Parylene-C provides an excellent barrier against the diffusion of water and the corrosion resistance [4] required by medical implants.

For this reason, Parylene-C has been widely used for the encapsulation of implantable devices [7,8,9,10,11]. There are, however, reports of progressive degradation of the polymer insulation barrier properties when it is exposed to saline solution in a body-mimicking environment [12,13].

Some authors suggested good blood compatibility of Parylene-C [14,15] but, up to date, its long term performance in the blood stream has not been fully investigated. Concerns have also been raised over the high susceptibility of the polymer to bacterial colonization [16].

Post-deposition exposure to radio frequency plasma is a straight-forward and versatile technique to modify the surface properties of Parylene-C without affecting its bulk properties. Chemically reactive ions bombarding the material are responsible for the physical modification of the surface and the formation of nanometer-sized features. The nature of the carrier gas used during plasma treatment influences the chemical composition of treated surface by introduction of free radicals. Hydrophilic Parylene-C coatings have been fabricated by O_2_ and air plasma treatment for anchoring drugs [17], or for improving cells adhesion in cell-based microdevices [14]. Super-hydrophobic Parylene-C layers have been successfully realized by consecutively exposing the polymer to low-power O_2_ and SF_6_ plasma [18,19]. This paper investigates the effects of plasma-induced nanostructuring of Parylene-C on its performance as an encapsulation material for blood contacting implants.

## 2. Materials and Methods

### 2.1. Samples Preparation and Plasma Treatment

Parylene-C was purchased, in dimer form, from Specialty Coating Systems, Indianapolis. Using a Parylene-C Labcoater series 300 (Plasma Parylene Systems GMBH, Rosenheim, Germany), a 10 µm thick layer of Parylene-C was deposited on two different substrates: glass and Polydimethylsiloxane (PDMS, Sylgard 184, Dow Chemical Company, Midland, MI, USA). Thin glass coverslips (1 cm × 1 cm × 0.17 mm) were preliminarily cleaned by sequential rinses with acetone, isopropanol and deionized (DI) water and dried on a hot plate One-millimeter thick PDMS slabs were fabricated by molding a thoroughly degassed mix of the polymer (Sylgard 184) and curing it on a at hot plate. An air plasma cleaning step was performed on all samples immediately before Parylene-C deposition.

The surface of the so prepared samples was nanostructured by consecutively exposing it to O_2_ and SF_6_ plasma. All treatments were performed in a reactive ion etching chamber (RIE-80, Oxford Instruments Plasma Technology, Bristol, UK) operating at the industrial standard frequency of 13.56 MHz. The chamber pressure during etching was maintained at 20 mTorr, with an RF power of 100 W and a gas flow of 100 sccm. The effect of different plasma exposure times was investigated.

### 2.2. Surface Characterization

The wettability of the nanostructured surfaces was evaluated in vitro by contact angle measurements. Two test fluids were used for this analysis: DI water and pig whole blood. The latter was obtained from the local abattoir and anticoagulated with citrate dextrose solution (ACD). ACD preserved the blood samples in liquid form for the required period, without significantly affecting its viscosity [20]. All contact angle measurements were performed within one hour from collection of the blood sample. The static contact angle was measured on the samples before and immediately after plasma treatment. A 3 µL droplet was released on the surface under test using a vertical syringe and digital images were recorded. The acquired pictures were analyzed using the software ImageJ to determine the contact angle. Each measurement was repeated three times, on three identically sized droplets. The reported data points are the obtained average of the static contact angles measured for the three droplets. The error bars represent the standard deviation.

The surface topography of Parylene-C coated glass slides was investigated by Atomic Force Microscopy (AFM) using a Park XE-100 AFM (Park Systems, Suwon, South Korea). Scans of 5 µm × 5 µm portions of the sample surface were acquired and the nanoscale roughness of treated and untreated samples was evaluated using the software Gwyddion (V2.50, gwyddion.net, Czech Republic).

### 2.3. Diffusion Barrier Performance

Two 3-inch glass wafers were cleaned in a piranha solution and covered with a 100-nm thick layer of sputtered copper (BAE 370, Balzers, Pfäffikon, Switzerland). The metal layer was patterned by photolithographic techniques in meanders with different line widths (50 µm and 100 µm) and overall length of 4 cm. The meanders were then coated with 5 µm of Parylene-C, and one of the two wafers was exposed to 10 min of O_2_ plasma followed by 1 min of SF_6_ plasma.

Afterwards, custom-made plexiglass containers were glued onto the wafers and filled with 0.9% saline solution, kept at 37 °C by means of a temperature controlled hotplate. The resistance of the copper meanders was monitored by a four-point measurement using a digital multimeter (34401A, Agilent Technologies, Santa Clara, CA, USA). A relay board was used to automatically switch every 5 min between the different samples under test.

### 2.4. Protein Adsorption

The adsorption of blood plasma proteins on treated Parylene-C was evaluated by fluorescence microscopy. For this analysis two proteins were considered: albumin and fibrinogen. 

At first, the individual adsorption of the single proteins was studied separately. Solutions containing 500 µg/mL of fluorescently marked albumin (Fluorescein isothiocyanate conjugate, Sigma-Aldrich, St. Luis, MO, USA) or 2.5 mg/mL of fibrinogen (Alexa Fluor™ 488 Conjugate, ThermoFisher Scientific, Waltham, MA, USA) in a saline buffer were used. Three 3 µL droplets of each solution were incubated for 1 h on the different substrates *(n = 3)*. During the incubation time, the samples were wrapped in aluminum foil to avoid degradation of the fluorescent marker. High humidity was maintained to prevent droplet evaporation. A similar procedure was used to investigate the competitive adsorption of the two proteins. For this test, two binary mixtures of albumin and fibrinogen were prepared. In each solution, only one protein at the time was fluorescently labeled. A ratio of 1:10 (*w*/*w*) of FBG/BSA was selected, similar to the ratio found in blood.

After incubation, all samples were rinsed with deionized water and dried under a nitrogen flow. Fluorescence images were acquired with an inverted microscope (TiEclipse, Nikon, Tokio, Japan) using the FITC ex465−495/em515−555 excitation/emission filter channel and analyzed using the software ImageJ. The average brightness is an indirect measurement of the amount of adsorbed protein. Images of the bare substrates were also acquired and used to compensate for the autofluorescence of the Parylene-C substrates.

### 2.5. In Vivo Testing

In vivo animal experiments were performed to evaluate blood-material interactions under physiological blood flow conditions. A 13 cm long commercial polyurethane sheath for arterial catheterization was modified to test the thrombogenicity of nanostructured Parylene-C in the blood flow.

To prevent bleeding and blood stagnation in the hollow catheter, the internal lumen of the sheath was entirely filled with PDMS. As a result, only the outer surface of the catheter was exposed to the blood flow. The catheter was coated with 10 µm of Parylene-C and exposed to 10 min of O_2_ plasma followed by 1 min of SF_6_ plasma. After sterilization, by means of ethylene oxide, the catheter was introduced in the left carotid artery of a sheep and left in place for eight days. Three Swifter-Charolais ewes between 47 and 51 kg were used for this test. The animals were sedated using ketamine and anesthesia was induced and maintained with isoflurane. A volume-controlled respirator (Julian, Drägerwerk, Lübeck, Germany) ensured mechanical ventilation of the animal after intubation. All hemodynamic parameters, were monitored throughout the study. This study was approved by the KU Leuven animal ethics committee (P228/2014). All animals received humane care in compliance with the ‘Principles of Laboratory Animal Care’ formulated by the National Society for Medical Research and the ‘Guide for the Care and Use of Laboratory Animals’ prepared by the Institute of Laboratory Animal Resources (National Institutes of Health). The status of the catheters during the experiment was monitored by non-invasive imaging techniques. Magnetic resonance images (MRI) of the neck of the animals were acquired three days after implantation (Magnetom, Trio Tim, Siemens Medical Solution, Erlangern, Germany). The flow rate of blood in the carotid artery was calculated from the MRI-derived images. Vascular ultrasound measurements were performed, immediately before explantation, to inspect for blood clots formation and verify the perfusion of the artery.

## 3. Results

### 3.1. Surface Wettability and Topography

Several studies report that the blood compatibility of a biomaterial can be altered by modifying its surface wettability and topography [21,22,23]. Plasma treatment is a widely used method to create stochastic nanostructures and to modify the surface roughness of Parylene-C [24,25,26] and other biomaterials [27,28]. 

The consecutive use of O_2_ and SF_6_ plasma to modify Parylene-C wettability was first proposed by Bi et al. [18]. In their study, the authors proved that the oxygen plasma pre-treatment determines an increase in surface roughness, while the following SF_6_ plasma is responsible for the injection of fluorine ions onto the surface. As a result, the surface energy and the wettability of the modified Parylene-C will vary strongly.

**Figure 1:**
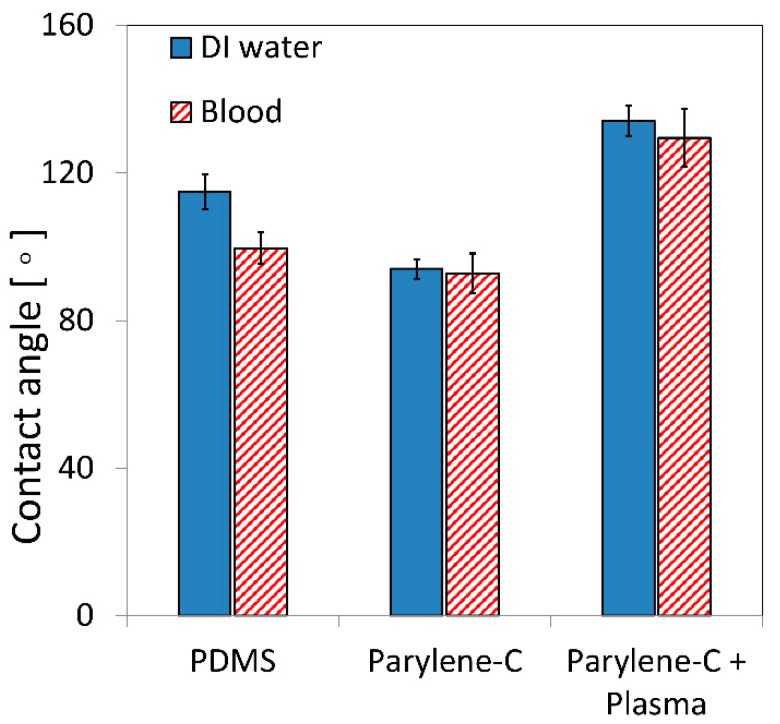
Measured contact angle for DI water and pig whole blood on Polydimethylsiloxane (PDMS) and Parylene-C before and after plasma treatment. The error bars represent the standard deviation between three different measurements.

At first, we investigated the effect of the proposed plasma treatment on the static contact angle of DI water and pig blood on Parylene-C. The results of this analysis are presented in Figure 1.

**Figure 2:**
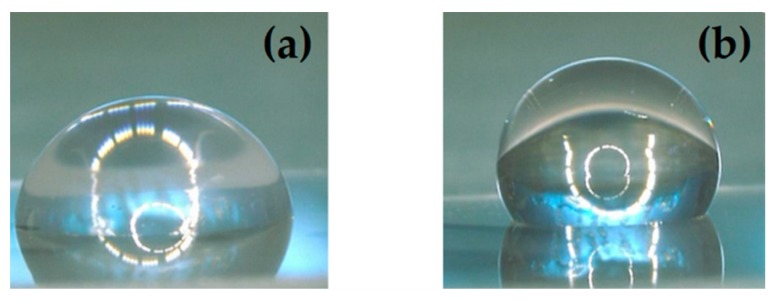
A 3 µL DI water droplet lying on the same Parylene-C coated surface before (**a**); and after (**b**) plasma treatment.

As a reference, the contact angles on PDMS were also measured and found to be 114.9 ± 4.8° for DI water and 99.6 ± 4.2° for blood. In agreement with what was reported by other authors [18,29], the contact angle of water, measured on untreated Parylene-C surfaces, was 86.7 ± 0.9°. After 5 min of O_2_ plasma pre-treatment, followed by 1 min SF_6_ plasma, the surface clearly manifested a more pronounced hydrophobic character. Figure 2 shows a water droplet lying on the same Parylene-C coated surface before and after treatment. As expected, the contact angles measured for pig blood were always lower than the ones measured for water, because of its lower value of surface tension with respect to water [30].

The influence of different O_2_ and SF_6_ plasma exposure times on the surface hydrophobicity was investigated.

**Figure 3:**
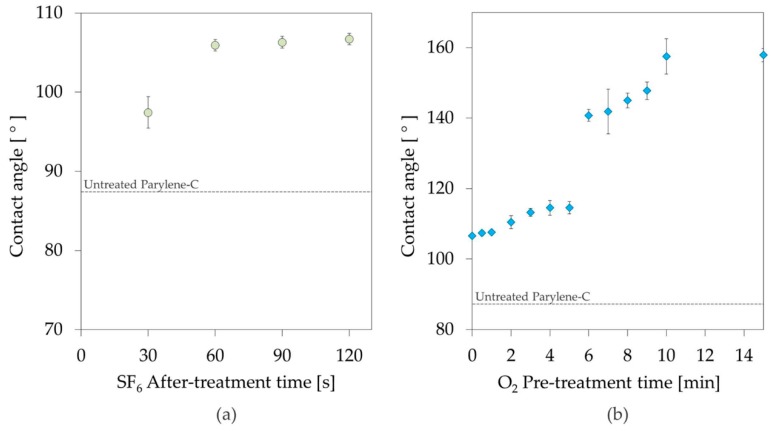
Static contact angle of DI water on Parylene-C coated glass slides as a function of: (**a**) SF_6_ after-treatment time (all samples were previously exposed to 30 s O_2_ plasma) and (**b**) O_2_ pre-treatment time (all samples were subsequently exposed to 60 s SF_6_ plasma). The error bars represent the standard deviation between three different measurements.

First, the duration of the O_2_ pre-treatment was fixed at 30 s and the duration of the SF_6_ after-treatment was progressively increased from 30 to 120 s. The results from this analysis are illustrated in Figure 3a.

In a second test, the duration of the O_2_ pre-treatment was progressively increased from 0 to 15 min, while the duration of the SF_6_ plasma after-treatment was kept at 1 min. The results of this study are presented in Figure 3b.

Figure 3b proves that the O_2_ treatment time has a greater influence on the surface wettability than SF_6_. Increasing the O_2_ pre-treatment time from 0 to 5 min resulted in an increase in the surface contact angle from 106.6 to 114.6°. When the treatment is prolonged more than 5 min the surface hydrophobicity drastically increases. A similar trend of the contact angle was observed and studied by other research groups [18]. The maximum contact angle measured for DI water was 158°, achieved after 10 min of O_2_ plasma followed by 1 min of SF_6_ plasma. No significant improvement was registered with longer O_2_ pre-treatments, up to 15 min.

The increased surface hydrophobicity of the plasma treated surfaces can be explained by mainly two factors: the physical roughening of the surface during the initial O_2_ plasma treatment, and the chemical modifications resulting from the introduction of fluorinated groups during the subsequent SF_6_ plasma treatment. As no substantial increase in the contact angle was registered when increasing the SF_6_ treatment time over one minute, we conclude that the surface is saturated with fluorine groups relatively quickly. On the other hand, the significantly higher hydrophobicity achieved with longer O_2_ plasma treatments, indicates a stronger influence of the physical roughening on the resulting surface properties.

**Figure 4:**
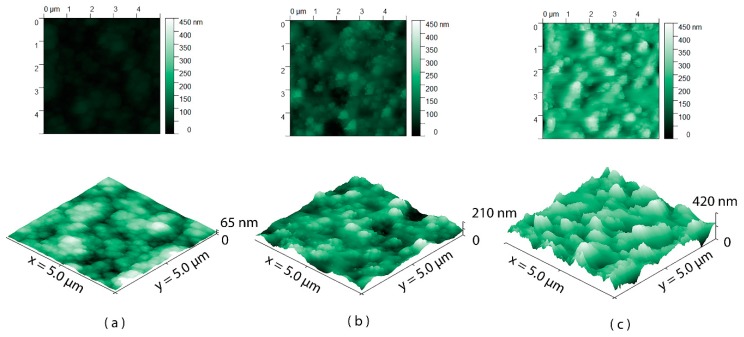
Atomic Force Microscopy (AFM) scans of (**a**) A1: untreated Parylene-C; (**b**) A2: Parylene-C treated with 5 min O_2_ and 1 min SF_6_; (**c**) A3: Parylene-C treated with 10 min O_2_ and 1 min SF_6_.

To quantify the increase in surface roughness induced by the oxygen plasma, AFM scans were acquired. As a reference, the surface roughness of the glass substrate was also measured. Table 1 gives an overview of the surfaces examined and the calculated surface roughness. The AFM scans of the Parylene-C surfaces are shown in Figure 4.

Inverting the order of the plasma treatments (SF_6_ pre-treatment followed by O_2_) failed to deliver super-hydrophobic surface, even when the O_2_ plasma was prolonged for as long as 20 min. On the contrary, the sample exhibited a high hydrophilicity, with DI water contact angles lower than 10°. This proves that the increase in surface roughness alone is not sufficient to overcome the effect of the OH radicals introduced by O_2_ plasma. In conclusions, the two treatment steps are necessary to fabricate super-hydrophobic Parylene-C surfaces.

### 3.2. Stability Study

The Parylene-C modification technique described above is intended for long term blood contacting implants. It is therefore important to verify that the surface properties do not degrade over time. A set of microscope slides was coated with a 10 µm thick layer of Parylene-C and submitted to the six different plasma treatments mentioned in Table 2.

**Figure 5:**
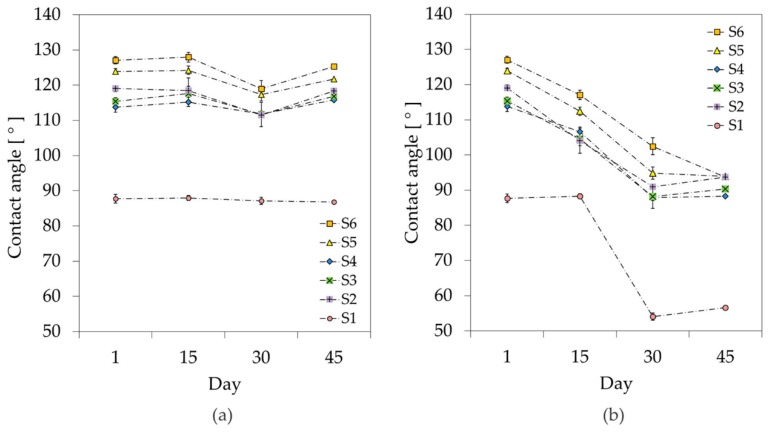
Long term stability study of plasma treated Parylene-C samples: (**a**) stored at room temperature and in dry environment; (**b**) stored in saline solution 0.9%. The error bars represent the standard deviation between three different measurements on three different samples.

To investigate how the storing conditions influence the samples hydrophobicity in the long term, and to reproduce possible effects of the body fluids on the surface properties, the test was performed by storing the glass slides in isotonic saline (0.9% NaCl solution). To reduce the influence of contaminants gathering on the surface under study, no samples were reused. Instead, dedicated samples were prepared for measuring the contact angle every 15 days and discarded after every test. The same experiment was conducted in parallel on a second set of slides prepared in the same run, but stored in a dry environment in a Petri dish. The static contact angles of the two batches of samples are plotted as a function of the storage time in Figure 5.

No appreciable change in the surface hydrophobicity was registered during the 45 days of measurement under dry storage conditions (Figure 5a). No substantial increase in the contact angle was registered between samples S2, S3 and S4, showing once again how the duration of the SF6 plasma only marginally influences the wettability of the treated surfaces.

When the glass slides are soaked in a saline solution, a significant decrease in the contact angle is evident over the investigated timespan (Figure 5b). This loss of hydrophobicity could be explained by delamination of the Parylene-C layer caused by the progressive infiltration of saline water in the few defects present in the film upon deposition. Moreover, it could be that the aqueous solution is responsible for the surface oxidation of the polymer. To identify the exact cause of the observed decrease in hydrophobicity, and to assess the long-term stability of the examined surfaces, a more thorough investigation is required (i.e., via X-ray photoelectron spectroscopy) and will be added in follow-up research. At the end of the experiment, the treated surfaces still exhibit a greater hydrophobicity than the untreated Parylene-C under the same storing conditions.

### 3.3. Diffusion Barrier Properties

One of the major concerns when Parylene-C is used as a packaging material for implantable devices is delamination of the polymeric film from the underlying substrate. This can result in the formation of pinholes and lead to infiltration of body fluids with the consequent failure of the insulating layer. Thoughtful substrate cleaning and polishing, together with the use of silane adhesion promoters (A174 SCS Inc., Indianapolis, IN, USA) are proven methods for improving Parylene-C adhesion and reducing delamination [31,32].

Previous studies [12] reported the formation of bubbles and delamination of thin Parylene-C films after just two days of soaking in saline solution. It is therefore important to investigate how the proposed plasma treatment influences the diffusion barrier properties of Parylene-C. For this purpose, a test setup similar to the one described by Op de Beeck et al. [13] was used.

**Figure 6:**
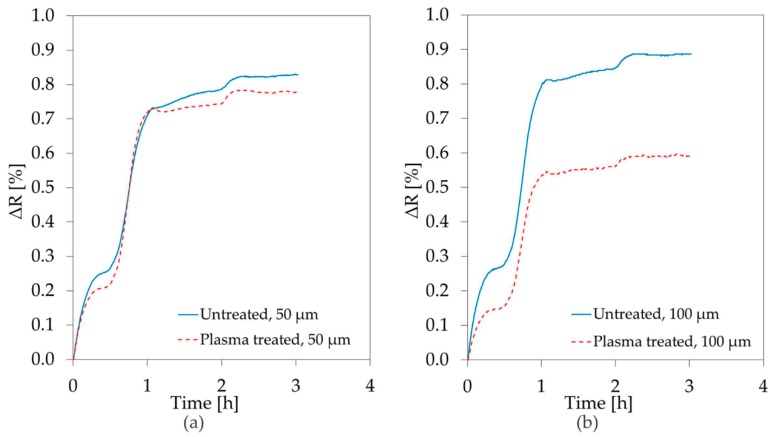
Percentage change, over 3 h, in the resistance of (**a**) 50 µm; and (**b**) 100 µm, wide Cu track exposed to saline solution 0.9% at 37 °C through a protective layer of 5 µm of Parylene-C.

Copper meanders were fabricated on glass, as discussed in Section 2.3, and protected by a layer of Parylene-C. The prepared samples were exposed to saline solution at 37 °C and their resistance was monitored. Diffusion of ions through the insulating layer will result in resistance changes due to corrosion of the subjacent metal. The results of the first 3 h of measurement are shown in Figure 6a,b, respectively for the 50 µm and the 100 µm line widths.

The percentage change in the resistance of the meanders underneath the as-deposited Parylene-C layer is presented with a continuous blue line, while the results for the plasma treated polymer are plotted with a dashed red line. Immediately after the beginning of the experiment, a minor resistance increase was registered for all the copper tracks. However, this increase slowed down after 30 min to then re-accelerate. This behavior, observed several times during the test, is widely known as healing, and it is due to cyclic formation and breakdown of protective layers of oxides on the surface of metal [33]. The smaller resistance increment measured for plasma treated Parylene-C, seems to suggest that this layer has better performances as a diffusion barrier. The same behavior was observed for both the examined line widths.

**Figure 7:**
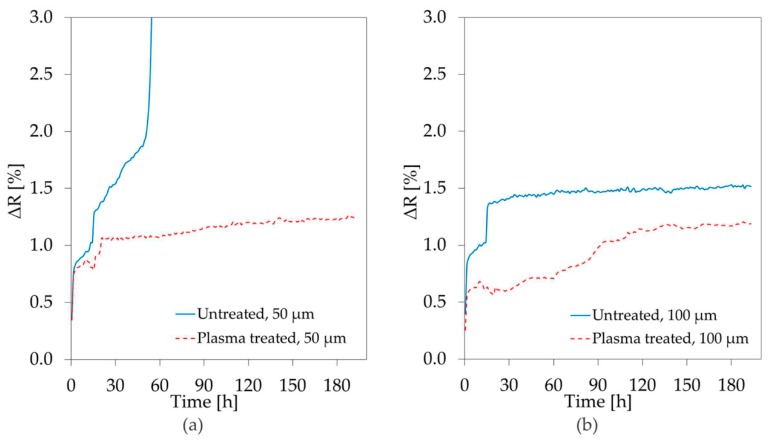
Percentage change, over eight days, in the resistance of (**a**) 50 µm; and (**b**) 100 µm, wide Cu track exposed to saline through a protective layer of 5 µm of Parylene-C.

Figure 7a,b present the evolution of the resistance values during approximately eight days of exposure to saline solution.

After 50 h the 50 µm wide meanders covered with untreated Parylene-C failed due to oxidation and infinite resistance was measured. The metal tracks protected by the plasma treated Parylene-C underwent a resistance increase of about 1% in 20 h, reaching a maximum of 1.2% increment after 200 h. Both samples with line width of 100 µm survived until the end of the test, but once again the smallest increase was observed for the plasma treated surface.

### 3.4. Plasma Protein Adsorption

Blood protein adsorption was examined as an indication of surface thrombogenicity. The adhesion and activation of platelets are complex processes mainly regulated by the protein film adsorbed on biomaterials upon contact with blood. Human serum albumin and fibrinogen are two of the most abundant proteins in the human blood and exert opposite effects on the thrombogenicity of a surface. HSA adsorption lowers thrombogenicity, being inert towards platelets receptors, and reduces both the number of adhered platelets and the degree of platelet activation [34,35]. FBG, on the contrary offers platelet-binding sites and is responsible for platelets activation and aggregation [36].

Several studies reported very effective adsorption of plasma proteins on Parylene-C surfaces [16,37,38]. Treating Parylene-C with an O_2_ plasma, yields hydrophilic surfaces and has been demonstrated to decrease albumin adsorption [14]. The consecutive use of O_2_ and SF_6_ plasma, yields superhydrophobic surfaces and will influence the adsorption of albumin and fibrinogen. In this study, we investigated the adsorption of single proteins and binary mixes of the two proteins by fluorescent microscopy.

**Figure 8:**
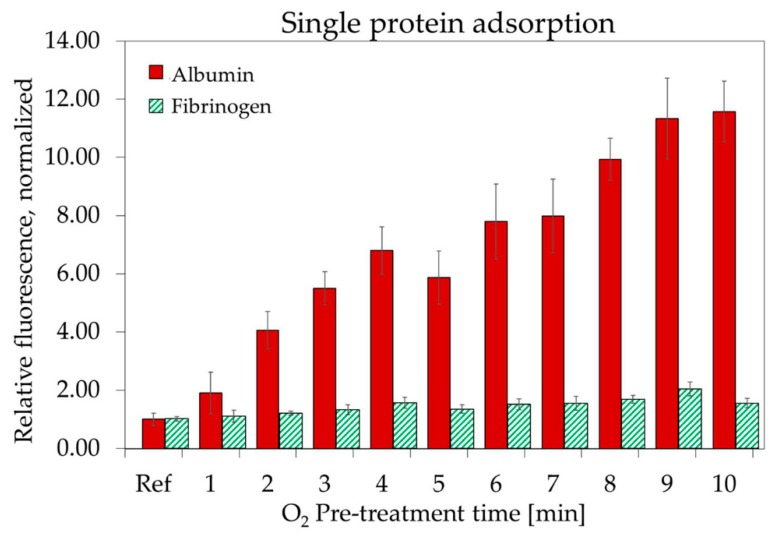
Single protein adsorption of albumin and fibrinogen on nanostructured Parylene-C surfaces in function of the O_2_ plasma pre-treatment time. All measurements are normalized to the adsorption measured, for each protein, on untreated Parylene-C. The error bars represent the standard deviation between three different measurements.

Figure 8 illustrates the adsorption of albumin and fibrinogen, in single protein solutions, in function of the O_2_ pre-treatment time. All samples were also exposed to a 1 min SF_6_ plasma. The bars in Figure 8 represent the relative fluorescence of Parylene-C surfaces after protein incubation. All data points relative to the same protein have been normalized to the fluorescence value for that specific protein, on untreated Parylene-C. Longer pre-treatment times resulted in higher adsorption of both proteins. This can be explained by the observation that proteins in aqueous solution more easily displace water from the hydrophobic surfaces to become adsorbed [39].

Increasing the O_2_ pre-treatment time from 1 to 10 min produced an 11-fold increase in the albumin fluorescence signal with respect to the untreated Parylene-C A much lower increase (2-folds) in fluorescence was registered, under the same conditions, for fibrinogen.

**Figure 9:**
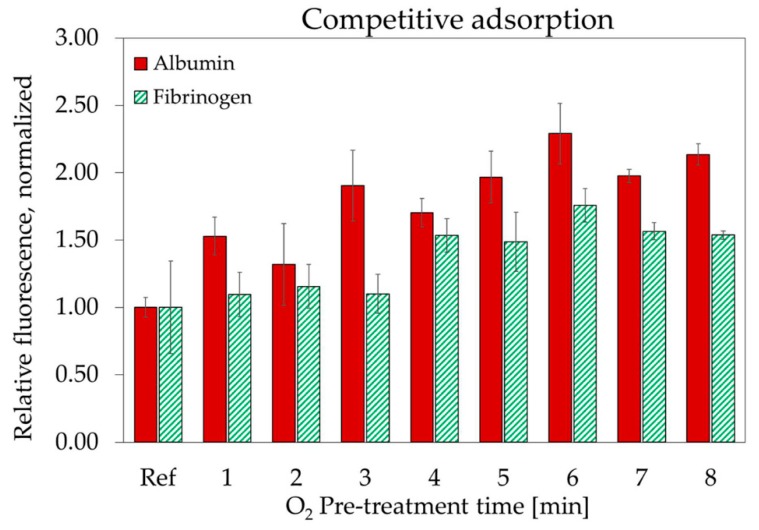
Competitive adsorption of albumin and fibrinogen on nanostructured Parylene-C surfaces in function of the O_2_ plasma pre-treatment time. All measurements are normalized to the adsorption measured, for each protein, on untreated Parylene-C. The error bars represent the standard deviation between three different measurements.

The competitive adsorption of HSA and FBG in binary solutions was also investigated. The results of this analysis are presented in Figure 9. As for the previous graph, all data points relative to the same protein have been normalized to the adsorption value of that specific protein, on the untreated surface.

While both proteins adsorbed more on the treated surfaces, the increase measured for HSA was significantly lower than for single protein solution. The presence of FBG in solution strongly outcompetes HSA, limiting its adsorption on the nanostructured surfaces. The adsorption of FBG, on the other hand, is less influenced by the presence of HSA. The total amount of adsorbed proteins increased significantly after plasma treatment. The larger increase in HSA adsorption was accompanied by a minor but non-negligible increase in FBG adsorption. This could be detrimental for the thrombogenicity of the nanostructured surfaces. However, some authors [3] suggested that not only the total amount of adsorbed FBG, but also its conformation can influence the thrombogenicity of a surface by exposing platelet-binding sites. All treated surfaces displayed a higher adsorbed albumin to fibrinogen ratio than the untreated samples. High HSA/FBG ratio is commonly associated to good haemocompatibility and low thrombogenicity [40,41], but it is difficult to predict how the higher amount of adsorbed HSA will influence FBG functional activity. Therefore, a more direct observation of the adhesion and the degree of activation of blood platelets is needed to confirm this finding.

### 3.5. In Vivo Validation

The nature of the protein layer adsorbed on a biomaterial gives a good indication of surface thrombogenicity. However, because of the many factors that govern the complex coagulation mechanism, these tests are not sufficient to demonstrate haemocompatibility [1]. Studying in vitro the adhesion of blood platelets and the degree of platelets activation can further support these conclusions.

**Figure 10:**
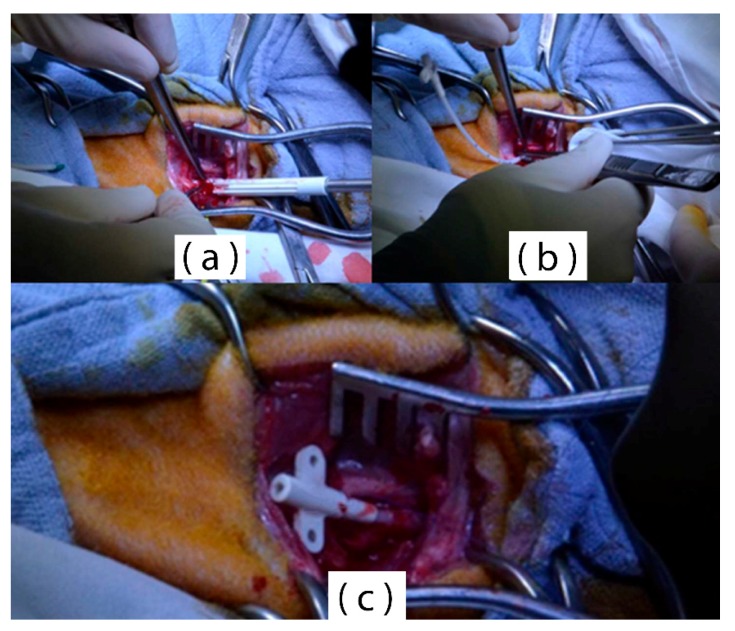
(**a**) Puncturing of the carotid artery; (**b**) insertion of the Parylene-C coated catheter; (**c**) catheter secured in the artery.

Platelets adhesion tests, under static or quasi-static conditions, fail to reproduce the exact physiological environment. Protein adsorption and platelet activation are largely influenced by the high shear stressed generated by the blood flow in a living organism. Hence, animal tests were performed to characterize the in vivo performance of the nanostructured Parylene-C surfaces. A cardiovascular polyurethane catheter was coated with Parylene-C and treated with 10 min O_2_ plasma and 1 min SF_6_ plasma. Three catheters were introduced in the left carotid artery of three sheep and exposed to the pulsatile arterial blood flow for eight days (Figure 10).

**Figure 11:**
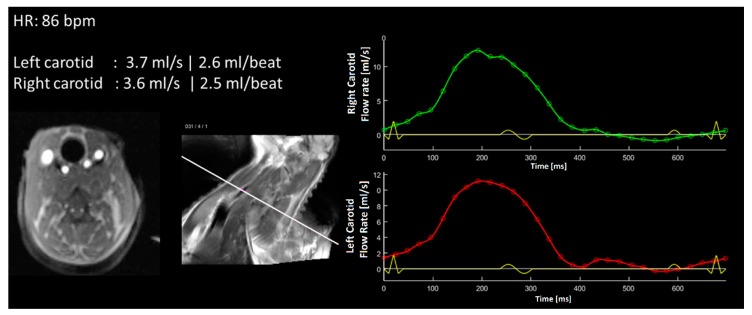
MRI scan of the neck section of one of the implanted sheep. Normal arterial flow and waveforms were recorded three days after implantation.

No anticoagulation therapy was administered for the duration of the test. Three days after implantation MRI images were obtained from the right and left carotids of each animal. No anomalies were detected, and normal values of volumetric flow in all vessels were recorded (Figure 11).

Echography scans of the animals’ neck were recorded eight days after implantation. No significant alteration of the carotid arterial flow, or adverse medical event, was observed. Only one of the three animals presented some fluid accumulation (seroma) at the incision point. After euthanasia, the section of the artery containing the implanted catheter was harvested. Both the surface of the catheter and the surrounding arterial vessel did not show any sign of clotting or thrombosis. The brain and all internal organs were inspected to rule out the possibility of embolus formations.

## 4. Discussion

The surface of Parylene-C was engineered in search of improving its haemocompatibility. The surface nanotopography of the polymer was modified by consecutive low power O_2_ and SF_6_ plasma treatments. The initial O_2_ plasma causes a physical roughening of the surface while the subsequent SF_6_ treatment lowers the surface energy of the polymer by the introduction of fluorinated groups. The combined effect of these physical and chemical modifications produces a highly hydrophobic surface. Replacing SF_6_ with other fluorinated gasses commonly used for plasma etching in microfabrication, such as CF_4,_ will very likely produce similar results. However, this has not been investigated in our study.

To study the degree of wettability as a function of the time dependent parameters of the process, the separate influences of O_2_ and SF_6_ were analyzed. An increase in the contact angle of water is registered when extending the duration of SF_6_ plasma that reaches its maximum after 1 min of treatment. From this we concluded that the surface is saturated with fluorine groups relatively quickly. More significant changes in the surface hydrophobicity are recorded when prolonging the O_2_ treatment for 10 min. Xiaopeng et al. [18] suggested that this behavior is the result of a transition from an homogeneous wetting (Wenzel model) to an heterogeneous wetting (Cassie–Baxter model) due to the increasing surface roughness.

The hydrophobic surfaces proved to remain stable up to 45 days from the treatment when stored at room temperature and in dry environment, with only minor variation in the measured water contact angle that can be attributed to contaminants gathering on the surfaces under test. However, when the samples were stored in isotonic saline solution for the same time interval, the degree of wettability changed significantly. Thanks to its high chemical bond stability, molecular weight and degree of crystallinity, Parylene-C is immune to the hydrolytic degradation that several biopolymers undergo when exposed to saline [42]. The reason for this decrease in water contact angle under wet storage conditions is therefore not clear, but could be explained by delamination of the Parylene-C from the underlying layer, and by propagation of defects and pinholes created in the film during the deposition process. Nevertheless, the untreated polymer manifested a similar behavior in the same time span, with a static contact angle that diminished by 30°. At the end of the measurement, the plasma treated surfaces still exhibited a higher hydrophobicity than the untreated counterparts.

The proposed treatment did not degrade the barrier properties of Parylene-C. On the contrary, when used to protect copper tracks from the corrosive action of normal saline solution at 37 °C, the treated polymer reduced the oxidation rate of the underlying metal better than as-deposited Parylene-C.

Excellent performances of nanostructured surfaces as a corrosion barrier have been reported in the literature [43]. When submerging superhydrophobic materials in saline solutions, an air film is captured between the nanostructures of the surface [44,45,46]. This trapped air film reduces the fraction of the surface effectively in contact with the fluid. As a result, the area for diffusion of corrosive Na^+^ and Cl^-^ ions through the insulation layer is also reduced. This observation could explain the better performances of plasma treated Parylene-C as a diffusion barrier.

Two of the most common blood proteins, albumin and fibrinogen, showed a higher affinity for Parylene-C surfaces after treatment. Water molecules are more easily removed from the solution/surface interfacial layer because of the higher hydrophobicity of the substrate. As a result, proteins are adsorbed in higher amounts. Changes in the surface topography also influence protein adsorption. There seems to be no general trend in the effect of textured surfaces on the amount of adsorbed protein [3]. Competitive adsorption tests revealed that, despite the limiting effect exerted by FBG on the adsorption of HSA, treated surfaces have a higher adsorbed HSA/FBG ratio, indicating lower thrombogenicity. 

However, this test alone cannot be considered conclusive for proving the improved blood tolerability of the material. Not only the ratio between albumin and fibrinogen adsorbed, but also the conformational changes undertaken by the proteins upon adsorption, can influence the degree of activation of adherent platelets. Moreover, albumin and fibrinogen are only two of the blood proteins involved in the coagulation process. In vitro studies of platelets adhesion and activation could support these conclusions, but are also only partially representative of the complex physiological response of a living organism.

Exposing the modified surfaces to the arterial blood flow of three sheep, during in vivo tests, did not cause any adverse event and did not trigger the coagulation cascade for the eight-day duration of the experiments. The experiment described demonstrates the short-term hemocompatibility of the nanostructured Parylene-C coating. Given that fibrinogen adsorption is still significant on the plasma-treated materials, more extensive in vivo studies will be performed to evaluate the long-term thrombogenicity of the proposed coating and compare it to untreated Parylene-C.

## 5. Conclusions

The chemical vapor deposition process of Parylene-C fabricates extremely thin continuous layers. The condensation of monomers from the gas phase, in a vacuum chamber, can be easily employed to deposit the polymer on a wide range of materials and in complex geometrical shapes. An RF plasma treatment is the only required process step to nanostructure the deposited polymer in a reliable and reproducible way. It influences both surface topography and chemical composition, yielding superhydrophobic surfaces with static contact angles as high as 158°. Plasma processes are relatively cheap, and applicable to a large variety of substrates.

The simplicity of the deposition process, and of the proposed modification technique, make it of great interest for coating implants chronically exposed to the blood flow, like vascular stents or blood pressure sensors for the direct measurement of arterial pressure [47], with complex geometries. Protein adsorption tests, and in vivo experiments suggest good blood tolerability of the treated material.

The extensive affinity of the treated surfaces to serum albumin, can also be exploited by pre-coating a dense blocking layer of the protein on the surface of an implant prior to implantation. This non-adhesive layer will prevent and limit the adsorption of other blood proteins.

## Figures and Tables

**Table 1:** Surface roughness measurement.

Sample	Substrate	Coating	O_2_ Plasma Duration (min)	SF_6_ Plasma Duration (min)	Surface Roughness (RMS) (nm)
A0	Glass	-	-	-	2 ± 1.2
A1	Glass	Parylene-C	-	-	10.96 ± 2.4
A2	Glass	Parylene-C	5	1	30.13 ± 6.2
A3	Glass	Parylene-C	10	1	53.48 ± 9.5

**Table 2:** Samples for stability measurement.

Sample	O_2_ Plasma Duration (s)	SF_6_ Plasma Duration (s)
S1	-	-
S2	30	30
S3	30	60
S4	30	120
S5	60	30
S6	120	30
